# Community-based monitoring as an accountability tool: influence on rural health services in Maharashtra, India

**DOI:** 10.1186/1753-6561-6-S1-O9

**Published:** 2012-01-16

**Authors:** Dhananjay Kakade

**Affiliations:** 1 Support for Advocacy and Training to Health Initiatives (SATHI), Pune, India

## Introduction

Introduction of comprehensive framework for community-based monitoring (CBM) and planning at various levels of the public health system has been one of the most significant policy initiatives under the national rural health mission. One of the key considerations while introducing this large-scale social experiment was to fill the accountability deficit of the public health system by involving local communities. In this paper I examine how CBM has contributed to improvement of local health services as well as some limitations of CBM.

## Methods

The act of tracking, recording and reporting the state of public health services in villages as experienced by people themselves is the core principle of CBM. In each monitoring cycle, the information is collected about the outreach services and the services of primary health centre at village level through group discussions. This is done by involving community members with emphasis on participation of women and other marginalised groups. Community responses are recorded in a form of rating each service as good, partly satisfactory, or bad. Information collected through this process is presented in a concise form as a ‘pictorial report card’ clearly reflecting availability, regularity, and quality of health services. These report cards rate health services using 11 indicators for which data is generated over a three-month recall period.

This paper is based on three rounds of CBM that took place in a period from July 2008 to December 2009 in the 220 villages spread over five pilot districts in western state of Maharashtra. Findings from these report cards were presented in the officially mandated public hearings (dialogues). Compiled data from different districts, reflecting the overall situation of health services, was presented to the state level health officials in the state level public dialogue, for further remedial actions.

## Results

At the beginning of CBM process, in the first round of monitoring exercise, on an average 48% of health services were rated as ‘good’ by community. This rating increased by 13 percentage points to 61% in round two, and by an additional 5 points to 66% in round three. The average percentage of services rated 'bad' by community decreased from 25% to 16% and to 14% over three subsequent cycles of monitoring.

We observed improvements in specific indicators e.g. immunisation improved by 21 percentage points from 69% rating it as 'good' in round one, to 90% rating it as ‘good’ in round three. Between the first and the third round of CBM, services by *anganwadi* (supplementary nutrition centre) and use of untied fund improved by 33 and 31 percentage points respectively. Health services by primary health centre (for example, 24-hour delivery services, in-patient services, laboratory and ambulance services etc.) improved from 32% in the first round to 74% in the third round.

While many aspects of the village health services improved, certain services like disease surveillance and village level curative services did not see much improvement. Status of these health services remained below 50% even after third round of CBM. Disease surveillance improved by only 9%, from 41% in first round to 50% in third round. Village level curative services remained at 42% in the third round compared to 40% in first round.

## Discussion

Improvements in health services witnessed over last three years seem to result from a combination of supply-side (health services) inputs and actions from demand-side (community) through CBM. However, CBM will have limitations in addressing systemic and structural issues, and may remain confined to the periphery of the public health system (i.e. outreach services or services by primary health centres) if it is not accepted as an accountability principle at all the levels of governance in the public health system. There seems to be a growing acceptance among health officials that community led action in form of CBM is integral to various strategies of the national rural health mission. However it certainly requires stronger reinforcement.

